# Olfactory Functioning and Depression: A Systematic Review

**DOI:** 10.3389/fpsyt.2017.00190

**Published:** 2017-09-28

**Authors:** Hannah Taalman, Caroline Wallace, Roumen Milev

**Affiliations:** ^1^Centre for Neuroscience Studies, Queen’s University, Kingston, ON, Canada; ^2^Department of Psychiatry, Queen’s University, Kingston, ON, Canada

**Keywords:** olfactory functioning, olfaction, depression, major depressive disorder, smell

## Abstract

**Background:**

Research has demonstrated a reduction in olfactory functioning in patients with schizophrenia. This research has led to examination of olfactory functioning in other mental disorders, such as depression. There is a great deal of variation in the results generated from such research, and it remains unclear as to how olfactory functioning is associated with or impacted by depression.

**Method:**

The current review examined the literature in accordance with PRISMA guidelines in order to generate a better understanding of this relationship and to identify if and what aspects of olfactory processing are altered. Through examination of the available literature from the databases PubMed, Ovid Medline, CINAL, and PsychINFO, 15 manuscripts were selected to determine if there was a difference in olfactory processing—specifically central and peripheral processing—between depressed individuals and non-depressed controls.

**Results:**

The comparison of the 15 studies showed that the majority of studies (9/15, 60%) found a difference in overall olfactory functioning between depressed individuals and non-depressed controls (*p* < 0.05).

**Limitations:**

There is still a lack of definitive conclusions due to variation of which olfactory process was altered.

**Conclusion:**

Given the differences in the methodology and design of these studies, a possible solution that could eliminate the lack of clarity and reduce variation would be to adhere to a single, thorough methodology that examines and separates central and peripheral olfactory processing. Future research employing a uniform and validated methodology could provide more definitive conclusions as to how and if olfactory functioning is related depression.

## Introduction

Within the global population, approximately 15% of the worldwide burden of disease is attributed to mental disorders (1, 2) and 8–12% will be affected by depression at least once in their life (3, 4). When focusing further on epidemiological factors, such as age and sex, the distribution of prevalence changes. The ratio of depression in women to men is typically cited as 2:1, with the rate of depression being higher in women (5). The prevalence of depression in certain age groups is higher in young adults (aged 18–44 years) relative to older adults, with sex differences occurring during these times (5). Depression is characterized primarily by low mood, decreased activity associated with reduced energy and fatigue, and loss of capacity for interest and enjoyment (anhedonia) (4, 6). Examination of the depressed brain has found a number of changes to various structures in the prefrontal limbic network (6), such as the orbitofrontal cortex, anterior and posterior cingulate cortex, hippocampus, amygdala, insula, and thalamus (3, 7). Many of these regions have projections connecting them to, or overlapping with, areas involved in olfaction (3). Recently, imaging studies have confirmed that emotions and odors are both processed in the hypothalamus, amygdala, orbitofrontal cortex, and the insular cortex (8–10). From an evolutionary perspective, one of the most primitive brain structures is the olfactory bulb (OB) which gave rise to the ancient limbic system responsible for emotional processes and contributing greatly to human survival (3, 11, 12).

Within the adult brain, the OB and hippocampus are the only areas that demonstrate postnatal neuroplasticity (13–15). Mouse models of chronic stress and previous research examining human hippocampal volume in major depressive disorder (MDD) have found a reduction in hippocampal volume and an abnormality in hippocampal neurogenesis (16–23). These changes are likely due to changes in glucocorticoid levels following chronic stress and may play a role in psychiatric disorders (16–23). Examination of this alteration suggests that use of antidepressant medication can restore some volume loss in the hippocampus, increasing the level of neurogenesis in this area (14, 15). The OB is a highly plastic structure with variations in the volume of the OB correlating with individual variations in olfactory functioning (13, 24–27). In the OB, a similar reduction in volume and neurogenesis has been observed. Animal studies have shown that OB volume decreases after periods of sensory deprivation and restores to normality after stimulation (25, 28). Through the use of rodent models, researchers have demonstrated that deficits in the OB impact the hippocampus and can induce a depressed mood by destroying/removing the OB. Song and Leonard (29) presumed that olfactory bulbectomy modulates behavioral responses by causing a dysfunction in the cortico–hippocampal–amgydalar circuit. Indeed, animal studies have shown that the depressive-like behaviors are likely attributed to changes in serotonin and dopamine levels (3, 29–32). Indeed, the OB specifically sends inhibitory projections to the amygdala [a structure that is preferentially involved in processing sadness and fear (31, 33)], and recent neuroimaging studies have found that in depressed patients and subjects of childhood maltreatment, a reduction in the OB volume is also observed (25, 31, 34). These rodent studies also found that the effects of the bulbectomy and the subsequent depression can be reversed with chronic use of antidepressant medication (30). Given these connections and the potential action of the OB in the cortico–hippocampal–amygdalar circuit, it is speculated that dysfunction at the level of the OB could potentially cause not only a reduction in olfactory functioning but also an increase in the number and severity of depressive symptoms (31, 35). However, in order to properly understand the implications of the results, it is first important to understand and differentiate the olfactory processing systems. Olfactory functioning can be divided into two types of processing: peripheral and central. Peripheral processing encompasses olfactory acuity, detection, and sensitivity, all of which is evaluated using measures of the olfactory threshold (1, 31, 36). Changes or deficits in the peripheral processing reflect impairments in processing at the level of the nasal epithelium, such as changes in the olfactory receptors (1). Central processing encompasses the cognitive processes associated with olfaction, such as identification, discrimination, memory, or the ability to label an odor (1, 31, 35–39). Central processing is measured using methods examining identification (the ability to identify and name an odor) and discrimination (the ability to distinguish between two different scents).

There is uncertainty regarding the connections observed, and if the therapeutic/restorative effect of antidepressant medication can be observed in the OB volume and neurogenesis of depressed humans. Based on findings previously observed in rodents, researchers have suggested that the use of antidepressant therapies including pharmaceutical and psychological interventions could also improve olfactory function (3, 34, 40, 41). However, there is much discrepancy within the literature as to how olfactory functioning is altered in depressed patients and an abundance of mixed results regarding what aspects, if any, of olfaction are impaired. While there is a wealth of studies available examining the olfactory perception deficits in schizophrenia (36, 37, 42–45), no conclusions have been generated with regards to other psychiatric disorders. The cognitive theory of depression proposes that MDD is associated with a negative bias in thinking, view of self, of experiences, of future plans, etc., and that this biased associative processing affects mental processes system wide—such as memory, attention, decision making, language, thought process, as well as executive and motor functioning (1, 46). Application of this theory with support from the previously established close neuroanatomical projections between the OB and the limbic system leads to the notion that these biases may also have an impact on sensorial functioning and olfactory processes in depressed individuals.

Despite the growing interest in this area of research over the last two decades, inconsistent results and no clear conclusion as to if and how olfaction is altered in depressed patients still remain. In order to more clearly understand the relationship between depression and olfaction, we have systematically examined the available literature and evaluated the olfactory data available comparing depressed patients to non-depressed controls. The goal of this study is to provide further understanding and overview of the studies that have been conducted over the last few decades with regards to this topic and to generate a conclusion with regards to the relationship between depression and olfaction.

## Methods

### Literature Search Strategy

**Figure 1:**
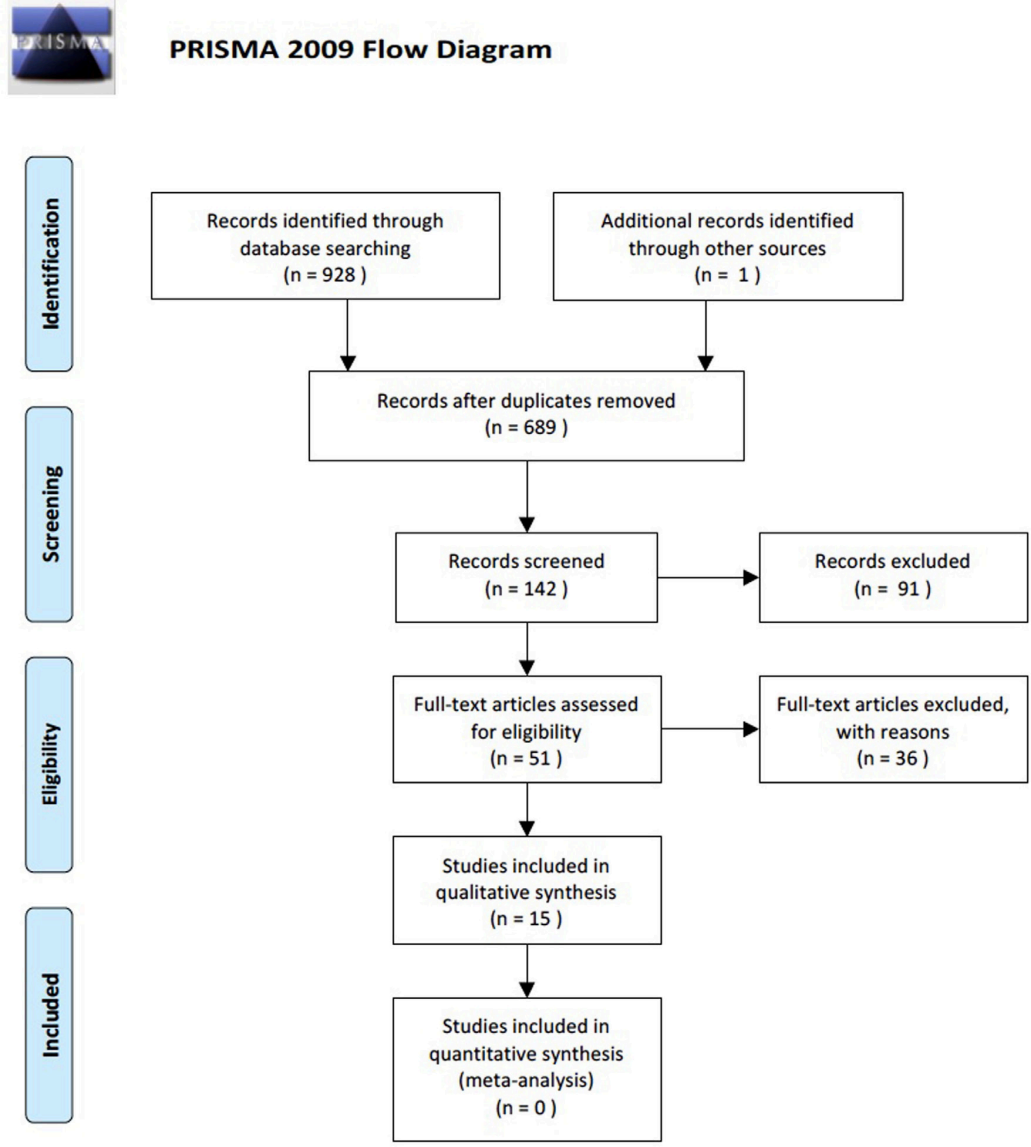
PRISMA flow chart outlining the number of details of the literature search with the total number of article gathered and the articles that met or did not meet inclusion criteria.

A search of the literature was conducted according to PRISMA guidelines (47) (see Figure 1) using the online databases PubMed, Ovid Medline, CINAL, and PsychINFO on May 25, 2016. The major search terms used were “olfactory functioning,” “anosmia,” “hyposmia,” “olfactory perception,” “sense organ disorder,” “smell,” “smell function,” “smell identification deficit,” “smell dysfunctions,” “smell detection,” “olfactory alteration,” “olfactory nerve diseases,” “olfaction disorders,” “odors,” “olfaction deficit,” “olfaction dysfunction,” and “depression,” “major depression,” “depressive disorder,” “*depression (emotion),” “bipolar disorder,” “depress*.Mp.,” and their related terms. Within the databases no date filters were applied but the literature was limited to English, those relating to humans, and not those with the key term “Parkinson’s disease.”

A total of 928 manuscripts were yielded from a search of the databases. These 928 studies were entered into EndNote™ Basic, where duplicates of the studies gathered were identified and eliminated to give a total of 698 manuscripts. The manuscripts were then reviewed using titles, with 142 manuscripts reviewed by abstracts, and finally 51 full manuscripts were reviewed. All titles, abstracts, and manuscripts were reviewed independently by two authors (Hannah Taalman and Caroline Wallace). All disagreements were discussed and reviewed by both authors until a consensus was reached based on inclusion/exclusion criteria. The references of the reviewed manuscripts were also examined for other studies of relevance. Of the full manuscripts reviewed, 17 were screened for inclusion/exclusion criteria with 15 meeting the criteria.

### Inclusion and Exclusion Criteria

For inclusion, all studies must have included data on a quantitative difference or loss of olfactory functioning in depressed and healthy control groups. All studies must have included subjects between the ages of 18 and 70, in order to exclude results due to age-related olfactory loss. Those studies that focused on primary olfactory dysfunction included patients with neurocognitive or neuropsychiatric disorders other than depression or employed patient self-report or subjective olfactory data were excluded.

### Quality Assessment

Each of the included studies were evaluated for quality in accordance with the Critical Appraisal Skills Program (CASP) criteria—a quality assessment tool for qualitative data in review studies that focuses on the following three elements: rigor, credibility, and relevance. The CASP is a 10-item questionnaire and the studies must meet all of the criteria in order to be included. 17 studies were examined under quality review based on these following questions: (1) clearly focused, (2) appropriate method, (3) cases recruited acceptable, (4) controls recruited in an acceptable manner, (5) accurate measurement and minimized bias, (6) confounds are addressed and accounted for, (7) results are precise, (8) results are believable, (9) can be applied locally, and (10) fir other available evidence. Of these 17 studies, 15 met criteria while 2 did not (25, 48). The study conducted by Amsterdam et al. (48) did not accurately minimize the bias in the study nor address or account for the confounds present in the study. Similarly, the study conducted by Croy et al. (25) did not meet the quality criteria as there was no accurate measurement and minimization of bias, the confounds were not addressed and accounted for, and the controls were not recruited in an acceptable manner. The remaining 15 studies met criteria, addressing each of the 10 questions asked by the CASP and were included in the review (see Table 1). One study conducted by Kopala et al. (42) did not directly specify the way in which they accounted for the confounds present and addressed in the study. However, the study was still included in the review as the potential confounds did not greatly impact the use of this manuscript in this review and all other major questions were addressed.

**Table 1:** Seventeen studies found relevant after full manuscript review were assessed for quality using Critical Appraisal Skills Program (CASP) criteria (CASP, 2013).

Trial	Clearly focused?	Appropriate method?	Cases recruited acceptable?	Controls recruited acceptable?	Accurate measurement and minimized bias?	Cofounds addressed and accounted for?	Results precise?	Results believable	Can be applied locally?	Fit other available evidence?	Include?
Amsterdam et al. (48)	✓	✓	NS	✓	χ	χ	✓	✓	✓	✓	χ
Atanasova et al. (8)	✓	✓	✓	✓	✓	✓	✓	✓	✓	✓	✓
Clepce et al. (49)	✓	✓	✓	✓	✓	✓	✓	✓	✓	✓	✓
Croy et al. (25)	✓	✓	✓	χ	χ	χ	✓	✓	✓	NS	χ
Croy et al. (3)	✓	✓	✓	✓	✓	✓	✓	✓	✓	✓	✓
Gross-Isseroff et al. (40)	✓	✓	✓	✓	✓	✓	✓	✓	✓	✓	✓
Hardy et al. (50)	✓	✓	✓	✓	✓	✓	✓	✓	✓	✓	✓
Kopala et al. (42)	✓	✓	✓	✓	✓	NS	✓	✓	✓	✓	✓
Lahera et al. (51)	✓	✓	✓	✓	✓	✓	✓	✓	✓	✓	✓
Lombion-Pouthier et al. (37)	✓	✓	✓	✓	✓	✓	✓	✓	✓	✓	✓
Naudin et al. (41)	✓	✓	✓	✓	✓	✓	✓	✓	✓	✓	✓
Negoias et al. (31)	✓	✓	✓	✓	✓	✓	✓	✓	✓	✓	✓
Pause et al. (35)	✓	✓	✓	✓	✓	✓	✓	✓	✓	✓	✓
Postolache et al. (52)	✓	✓	✓	✓	✓	✓	✓	✓	✓	✓	✓
Swiecicki et al. (53)	✓	✓	✓	✓	✓	✓	✓	✓	✓	✓	✓
Warner et al. (54)	✓	✓	✓	✓	✓	✓	✓	✓	✓	✓	✓
Zucco and Bollini (55)	✓	✓	✓	✓	✓	✓	✓	✓	✓	✓	✓

*NS, not specified*.

### Data Extraction and Statistical Analysis

HT extracted the data from those studies meeting criteria. The information gathered was as follows: number of cases and non-depressed controls; methodology employed for measurement of olfaction and the specific aspect of olfaction focused on; demographics; correlations, means, SDs, and conclusions regarding depression and olfaction; as well as any other relevant or pertinent information.

## Results

### Olfactory Functioning in Depressed Compared to Control

Table 2 provides a simplified summary of major aspects of the studies for comparison of olfactory aspects examined, olfactory measures used, and summation of those that had significant results (for full review, see Table S1 in Supplementary Material). Of the studies examined, eight studies included some measure of olfactory threshold (3, 31, 35, 37, 40, 48, 50, 52). Of these studies, half (four) found no significant difference between depressed patients and non-depressed controls with regards to threshold scores (*p* > 0.05, see Table S1 in Supplementary Material for specific statistical values) (3, 35, 48, 50). The remaining half found a significant difference (*p* < 0.05, see Table S1 in Supplementary Material for specific statistical values) between the two groups, with depressed scoring significantly lower than controls (31, 37, 40, 48).

**Table 2:** Summary of key aspects of each of the included studies.

Studies	*n*	Age range and gender ratio	Threshold	Discrimination	Identification	Depression measure	Olfactory test(s)	Significant difference	Aspect of difference
Atanasova et al. (8)	30 depressed	Age range: not stated; mean age: 34.6		x	x	DSM-IV	Two odorants (vanillin and butyric acid) at 3 concentrations each, 9 combination of the odorants and 2 control	Yes	Hedonic
	30 controls	Gender ratio: 12 females, 18 males				MADRS			

Clepce et al. (49)	37 current depressive episode	Age range: 23–71; mean age: 47.52			x	DSM-IV	Sniffin’ Sticks	Yes	Identification
	17 remitted	Gender ratio: 21 females, 16 males				BDI			
	37 control					SHPS			

Croy et al. (3)	27 depressed (female only)	Age range: 22–59; mean age: 38.5	x	x	x	BDI	Sniffin’ Sticks	Yes	Discrimination
	28 control (female only)	Gender ratio: 27 females, 0 males				HAM-D			

Gross-Isseroff et al. (40)	9 depressed	Age range: 34–67; mean age: 49.0	x			DSM-III-R	Three-way forced choice of target scent at different concentrations	Yes	Threshold
	16 controls	Gender ratio: 8 females, 1 male				HAM-D			

Hardy et al. (50)	20 bipolar disorder	Age range: 20–53; mean age: 31.1 (males), 35.6 (females)	x		x	DSM-IV	STT	No	
						DIGS			
	44 control	Gender ratio: 15 females, 5 males				PANSS	University of Pennyslvania Smell Identification Test (UPSIT)-40		
						YMRS			

Kopala et al. (42)	21 depressed	Age range: 21–56; mean age: 37.0			x	DSM-III-R	UPSIT-40	No	
	77 control	Gender ratio: 13 females, 8 males							

Lahera et al. (51)	39 euthymic bipolar disorder	Age range: 18–70; mean age: 46.82			x	DSM-IV-TR	UPSIT-40	Yes	Identification
	30 control	Gender ratio: 22 females, 17 males				HAM-D			
						YMRS			

Lombion-Pouthier et al. (37)	49 depressed	Age range: 20–60; mean age: 43.4	x		x	DSM-IV	Test Olfactif	Yes	Threshold
	58 control	Gender ratio: 35 females, 14 males				BDI			

Naudin et al. (41)	18 depressed	Age range: 20–74; mean age: 50.1		x	x	DSM-IV	Two odorants at different concentrations and different combinations	No	
	18 clinically improved	Gender ratio: 12 females, 6 males				MADRS			
	54 controls								

Negoias et al. (31)	25 depressed	Age range 21–55; mean age: 36.86	x	x	x	DSM-IV	Sniffin’ Sticks	Yes	Threshold
	22 control	Gender ratio: 17 females, 4 males				BDI			

Pause et al. (35)	24 depressed (18 participated at time 2)	Age range: not stated; mean age: 48.4	x			DSM-IV	Threshold was obtained using two odorants in a staircase detection procedure	No	
	24 control	Gender ratio: 15 females, 9 males				BDI			

Postolache et al. (52)	14 seasonal affective disorder (SAD)	Age range: 27–66; mean age: 42.3	x			DSM-IV	Phenyl ethyl alcohol presented in a staircase paradigm	Yes	Threshold
	16 control	Gender ratio: 7 women and 7 males				SIGH-SAD			

Swiecicki et al. (53)	20 recurrent depressive disorder	Age range: 18–70; mean age: 38.2	x		x	DSM-IV	Sniffin’ Sticks	No	
	21 bipolar affective disorder					HAM-D			
	5 first lifetime episode of depression	Gender ratio: 30 females, 16 males				AUDIT			
	30 control					BDI			

Warner et al. (54)	6 depressed	Age range: 38–50; mean age: 37.0			x	RDC	UPSIT-40	No	
	8 control	Gender ratio: 0 females, 6 males							

Zucco and Bollini (55)	12 mild depressed	Age range: 23–58; mean age: 41.3			x	DSM-IV	Odorants presented with 4 verbal options for naming the target	Yes	Identification
	12 severe depressed	Gender ratio: 12 females, 12 males							
	12 control								

*DSM, Diagnostic and Statistical Manual; HAM-D, Hamilton Depression Rating Scale; MADRS, Montgomery–Asberg Depression Rating Scale; BDI, Beck Depression Inventory; SHPS, Snaith-Hamilton Pleasure Scale; DIGS, Diagnostic Interview for Genetic Studies; PANSS, Positive and Negative Syndrome Scale; UPSIT-40, University of Pennsylvania Smell Identification Test, 40-item test; YRMS, Young Mania Rating Scale; STT, Smell Threshold Test; SIGH-SAD, Structured Clinical Interview Guide for the Hamilton Depression Rating Scale, SAD version; AUDIT, Alcohol Use Disorders Identification Test; RDC, Research Diagnostic Criteria*.

*Significant difference in all cases indicated a poorer olfaction score for depression when compared to non-depressed controls. No significant difference indicates scores between depressed individuals and non-depressed controls had little to no difference*.

Only four studies examined olfactory discrimination (3, 8, 31, 41). Of these studies, only Croy et al. (3) found a significant difference between depressed and non-depressed controls at baseline measurement (*p* = 0.037) with depressed scoring lower on the discrimination tests. No significant difference after a psychotherapy program. The remaining studies did not find any significant difference in the olfactory discrimination scores in depressed compared to control.

The aspect of olfaction most commonly examined was olfactory identification, with a total of twelve studies including a measure of olfactory identification in the methodology (3, 8, 31, 37, 41, 42, 48, 49, 51–54). Four of twelve studies found a significant difference between depressed and non-depressed controls with the identification scores being lower in depressed than controls (*p* < 0.05) (8, 49, 51, 53). The remaining found little to no difference between the scores of depressed and control.

Methodology used for the measurement of olfactory functioning varied. Olfactory parameters were measured using three testing paradigms: Burghart’s Sniffin’ Sticks, University of Pennyslvania Smell Identification Test (UPSIT), and the use of varying concentrations and/or a variety of chemicals with differing odors. Four of the fifteen manuscripts employed the Sniffin’ Sticks Extended Test to measure threshold, discrimination, identification, or a combination of any of those three (3, 31, 52, 53). Another four used the UPSIT to measure the identification ability (42, 48, 49, 51). The remaining seven measured olfaction by using a number of odors to measure identification or at least one odor presented in a staircase paradigm to measure threshold.

## Discussion

### Olfactory Functioning Changes in the Examined Literature

A lack of understanding of the relationship between olfaction and depression is seen throughout the literature for many different aspects of olfactory functioning. The goal of our study was to examine the available literature pertaining to the topic of olfactory dysfunction in individuals with a primary diagnosis of depression, including major depression disorder, seasonal affective disorder (SAD), and bipolar depression (BD)/disorder. A comparison of the fifteen studies which met criteria shows that nine of fifteen (60%) studies found significant differences between depressed and non-depressed controls on some aspect of olfactory functioning, with depressed having scores that reflected poorer olfactory functioning. However, the remaining six found there was no significant difference at baseline between depressed and non-depressed controls. Upon further examination of the results, it is important to note that while the results of some are not significant, in some studies the depressed patients performed poorer than non-depressed controls on average (3, 52). An overall examination of these studies and a combination of the results provides a better picture of the relationship between olfaction and depression, and further evidence to support that a reduction in aspects of olfactory functioning may be associated with current depression.

The aspect of olfactory functioning with the most agreement with regards to a significant difference between depressed and non-depressed controls was the aspect of olfactory threshold, also termed as olfactory sensitivity in some studies. Four of the eight studies that examined threshold found that was a significant difference between the olfactory threshold of depressed and that of non-depressed controls (3, 35, 48, 52). All of these studies found that the threshold was lower in depressed participants than the non-depressed controls. Next, identification was the most commonly studied aspect of olfactory functioning and four of the twelve studies examining this aspect of olfaction found significantly lower identification scores in depressed compared to non-depressed controls. While they did not find a significant difference between depressed and non-depressed controls, Croy et al. (3) found that the mean identification score was lower in patients (mean = 26.7, SD = 3.4) compared to non-depressed controls (mean = 27.2, SD = 3.4) at baseline. While there was little to no difference between these scores and this difference being less than 1.0, the lower means do suggest that with a larger sample size—which in the case of this study was 27 females with depression and 28 healthy females—there may be a greater or a statistically significant difference between these two groups. Of the fours studies examining discrimination, only one study (3) found a significant difference between depressed and non-depressed controls. Overall, four studies found an alteration in threshold and, therefore, peripheral processing, and four studies found an alteration in discrimination and identification meaning an alteration in central processing. While there is an even split between which processing aspects of olfaction are altered, we indeed conclude that there is an alteration in olfactory functioning in depression. Although some studies examine aspects such as intensity, hedonics, etc., we focused only on threshold, discrimination, and identification given their clear roles in and separation into the olfactory processes.

### Olfactory Functioning and the Treatment of Depression

One aspect of the literature that was of particular interest was the alteration in olfactory functioning before and after treatment for depression. Of the fifteen studies examined, only four used an experimental design involving an interventional protocol of either antidepressant medication (40, 53), psychotherapy (3), or a combination of the two (35). Croy et al. (3) examined the differences between olfactory scores in depressed before and after psychotherapy, comparing this to non-depressed controls with use of the Sniffin’ Sticks testing paradigm. The researchers found lower odor scores on all aspects of olfaction (threshold, discrimination, and identification) with only discrimination being significantly lower in depressed than non-depressed controls. After psychotherapy, no significant difference on any aspect of olfactory functioning was observed; suggesting that the successful treatment of depression also treated the olfactory dysfunction. Pause et al. (35) found a strongly, though not significantly, reduced threshold in depressed before the initiation of a combination of psychotherapy and antidepressant medication, and the olfaction scores strongly correlated with the depression score of the Beck Depression Inventory. After treatment, neither significant differences nor any correlations were observed between the depressed and control groups. Clepce et al. (49) and Gross-Isseroff et al. (36) both focused their study on antidepressant medication as the intervention and found significant improvements in the olfaction scores after treatment. Clepce et al. (49) employed the Sniffin’ Sticks methodology to measure identification, finding significantly lower identification scores in depressed at baseline compared to non-depressed controls and within the group after treatment with antidepressant medication. No significant difference was found between remitted and non-depressed controls. Gross-Isseroff et al. (40) employed a methodology focusing on threshold scores for two odorants at varying concentrations. These researchers found no significant difference between depressed and non-depressed controls at baseline or 3 weeks after initiation of treatment; however, 6 weeks after the initiation of treatment, a significant improvement in sensitivity to one target scent of isoamyl acetate was found. These studies are of particular importance and interest as they help to address whether an improvement in depression can also cause an improvement in olfactory functioning when there is a dysfunction at baseline. The results of which lead to the belief that the treatment of depression can improve not only the depressive symptomology but also the olfactory dysfunction associated with such. Further studies are required to confirm the findings of an improvement in olfactory functioning after treatment and to provide further evidence of a baseline dysfunction. However, these four studies represent an excellent point from which further research and understanding can develop.

### Limitations in the Literature

Overall, we do indeed find that olfactory functioning is impaired in depression; however, the variation in what aspect of olfaction is impaired remains mixed. Kohli et al. (12) suggests that the variations in the results available in the literature may be due to different patient populations, variance in the olfaction measures, small patient cohorts and small sample sizes, and some studies using patients with primary olfactory dysfunction while others use patients with primary depression. Indeed, of the fifteen studies we examined there was a great deal of variation in the olfactory testing procedure employed—with the most common being Sniffin’ Sticks (27% of the studies employed this test) and UPSIT (20% of the studies employed this test)—and the aspect of olfaction measured. The remaining studies examined olfactory functioning using a self-made olfactory test. Additionally, very few studies examined all three of the main aspects of olfaction—threshold, discrimination, and identification. Only two studies (3, 31) examined all three of these aspects and, therefore, were able to properly compare the changes in olfaction. These studies employed Sniffin’ Sticks to measure olfaction and used the extended test to examine the main aspects of olfaction but differed in number of times measured, gender of participants, and if an intervention was included in the protocol. Both found a significant difference between patient and control at baseline with regards to one aspect of olfactory functioning. In the case of Negoias et al. (31), there was a significant difference between threshold of depressed patients and non-depressed controls, with patients demonstrating a lower threshold score. Croy et al. (3) found a significant difference between the discrimination ability of patients at baseline; however, these researchers measured olfaction before and after the use of a depression intervention with no significant differences occurring after treatment. These two studies, while finding a change in central processing by one (3) and peripheral processing by the other (31), represent the starting point for development of further research within this field.

Many studies examined only one or two of threshold, discrimination, or identification using either the UPSIT, Sniffin’ Sticks or a lab-made procedure. The variation within the testing procedures likely contributes to the inconsistency in the literature and further hinders the formation of a clear conclusion as to the nature of the relationship between olfaction and depression. We assert that a solution to this issue is tangible and obtainable by researchers adhering to a single, consistent methodology that involves testing all three of: threshold discrimination, and identification. Measuring all three aspects of olfaction allows or differentiation of the processing systems and closer examination of which, if not both, systems are impacted in depression.

The majority of the studies available in the literature examine olfaction at only one time which may be the etiological or contributing factor for the variation and confusion as to the relationship between depression and olfaction. While some researchers find no relationship between the two as there is no significant difference between non-depressed controls and depressed patients, many of the patients were already under treatment and how the olfaction changes at the initiation or over the course of this treatment was not examined. Observation of how olfactory processing, especially central processing, is altered in conjunction with treatment of depression could further substantiate and provide evidence for the theory that OB volume and neurogenesis restoration can be observed in depressed humans after improvement of depression severity. The improvement of depression severity is believed to be linked to an increase in neurogenesis in the hippocampus (14, 15) and this theory could be applied to the OB—which is also a site for neurogenesis in the adult brain—by examining how changes in one affect the other and the relationship between the hippocampus, OB, and their associated processing systems.

In addition to inconsistency in the methodology employed and small sample sizes, the age range of the participants may have an impact in the results. All of the studies included participants within the age range of approximately 20–70 years, with some variation in the exact range throughout (see Table S2 in Supplementary Material for full review with age mean and range). We included studies with participants in this age range in order to control for age-related decline in sensory functioning and to focus primarily on adults rather than elderly individuals. While this would allow for a more inclusive study, the maximum age still poses a specific problem with regards to the impact of aging in olfactory functioning. In particular, the inclusion of participants over the age of 60–65 and the comparison of the olfactory functioning of such participants to those younger represents a limitation and a possible explanation for the variation in the results and a lack of significance. A decrease in olfactory functioning is a common occurrence and consequence of aging with age having a strong influence on the results of psychophysical tests of olfaction (56). Within the aging population, the prevalence rate of olfactory dysfunction is 13.9% in individuals 65 years old, with this rate rising to over 50% in individuals between 65 and 80 and 80% in those over 80 years of age (57–60). The increase in olfactory dysfunction prevalence in those between the age of 65 and 80 may be interfering with the results of those studies examining olfaction in depressed individuals, particularly those with higher mean ages and individuals closer to the range where olfactory dysfunction is due to age, such as Clepce et al. (mean: 47.52) (49), Gross-Isseroff et al. (mean: 49.0) (40), Naudin et al. (mean: 50.1) (41), and Pause et al. (mean: 48.4) (35). By including individuals over the age of 60, researchers are increasing the potential that the olfactory dysfunction observed in an individual may be due to the general age-related decline in olfaction rather than due to depression. This would not allow for proper examination of the disease-state of depression and introduces an important variable that could not be eliminated without further limiting participant criteria. Therefore, we propose further limitations on the age of the participants in order to reduce the possibility of olfactory dysfunction due to age. Given that age-related decline is examined in those approximately 65 and older, we propose limiting the participant age range to those between the age of 18 and 60. This limitation would allow for further isolation of olfactory functioning due to depression and a reduction in the number of confounds or comorbidities that could be influencing the results and contributing to the lack of clarity within the literature.

### Systematic Review and Meta-Analysis in the Literature

Within the literature, there are very few reviews on the topic of olfactory functioning and depression. However, of those that have been published, a recent publication by Kohli and colleagues (12) focusing on this topic provides an excellent outline of the available research and valuable meta-analysis. While this publication is very similar in topic and included similar literature as ours, there are a number of differences between the two. First, Kohli et al. (12) included literature that focused on primary olfactory dysfunction studies and the associated depressive symptoms. For the purpose of our study, we included only those manuscripts focusing on depression as the primary diagnosis/disease for inclusion in the qualitative and quantitative analysis. Second, the researchers focused primarily on MDD while we included not only MDD but Bipolar Disorder/BD and SAD. Third, we placed further limitations on the study populations by using only studies where the age range of the population was between 18 and approximately 70. We, therefore, eliminated any studies that focused primarily on the olfactory functioning in elderly patients; which in itself warrants a separate review given the availability in the literature. Finally, of the 15 articles included in their main table (12) (see Table S1 in Supplementary Material) we included 8 of the same articles in our table with an additional 7 not included by Kohli et al. (12).

### Limitations

While conducting the literature review and analysis of the results, we encountered a number of limitations. The largest limitation we encountered was during the meta-analysis phase of the review. We intended to conduct a meta-analysis of the four studies that examined olfaction before and after treatment, with the goal of finding a common conclusion through the combination of the data. However, we were unsuccessful in doing so due to the variable methodology and reporting employed by the researchers, the manner in which the data were analyzed and presented by the researchers, and the small number of studies included in the analysis. Of the four studies, only two used the same methodology (3, 53), while the other two employed an alternative method (35, 40). The variation in the methodology employed did not facilitate a standardization of the results in such a way that could be combined and compared properly. This particular limitation could be addressed and eliminated in further reviews if researchers adhere to a global methodology for assessment of olfactory functioning, such as the methodology we have suggested. The use of differing methodologies also meant that the manner in which the results were presented was different throughout; the combination of the different results further hindered our ability to conduct a meta-analysis. As well, the number of articles that were to be included in the meta-analysis was too small. That posed further issue given the lack of available and applicable data, as well as a potential skewing of the data given the small sample sizes in each. With further development and expansion of research focusing on the change of olfactory functioning before and after treatment for depression, this particular limitation could be resolved and a proper meta-analysis conducted in the future. Another limitation we encountered throughout was the vast amount of contradictory evidence. While the majority (60%) of the fifteen articles we examined found a significant difference between depressed individuals and non-depressed controls on some aspect of olfaction, there remains mixed results as no one aspect of olfaction was found to be consistently different. This did not allow us to make any specific conclusions, having to generate a broad conclusion instead.

### Clinical Implications

In assessment of depression, particularly at the onset of depressive episodes, many clinical scales—such as the Montgomery–Asberg Depression Rating Scale and Hamilton Depression Inventory—measure a loss of appetite as an indicator of hedonic evaluation. However, the underlying mechanisms and representation of hedonics in the sensory system are not measured further (49) and often not investigated beyond a handful of questions. Although the results of the literature remain mixed as to which aspect of olfaction is altered, the presence of a general alteration warrants further questioning and examination at the clinical level in order to better understand the individual experience of depression and the associated sensory hedonics. Integration of measurement of olfactory functioning of the individual at first examination and throughout treatment could allow for a more thorough perspective of severity and progression of the individual depressive episodes. Given the findings of Clepce et al. (49), Croy et al. (3), Gross-Isseroff et al. (40), and Pause et al. (35), the measurement of olfactory functioning throughout treatment could provide an additional measure of treatment response and success. Overall, the integration of further sensory measures would allow for a better understanding of the underlying mechanisms of hedonics and the individual experience of depression.

Olfaction is a sense that individuals often take for granted and do not notice when there is a reduction in their ability to smell. However, olfaction is greatly involved in quality of life through ability to enjoy surroundings, involvement in appetite and taste quality of food, and heavily integrated into memory storage and recall (31, 61). Examination of primary olfactory dysfunction has demonstrated the impact of lack of sense of smell on the ability to enjoy preparing food and the affective experience associated with eating, as well as detecting spoiled or inedible food (3, 12). Applying this research to a depressed population suggests that the loss of appetite and motivation to eat in depressed might be due in part to a loss of overall olfactory functioning. In addition to the nutritional deficits, this loss of appetite and anhedonia associated with appetite and eating also plays a role in the reduction of socialization of depressed individuals. Aside from the basic biological role, the experience of eating often has a larger social role (12, 62) that would likely cause further anxiety and be unappealing to an individual who already is experiencing difficulty with motivation, loss of enjoyment of food, loss of enjoyment of socialization and an association social withdrawal, and fear of stigmatization. The social pressure associated with food likely would cause further withdrawal from social situations and further loss of appetite, avoidance of food, and further depressed mood. A greater understanding of the relationship between olfaction and depression at the clinical level provides a better overall picture of the manner in which quality of life is affected. Integrating this research into clinical practice to identify deficits and adjust by building and focusing on a sensory rich environment—particularly with regards to foods and smells that have positive memory associations—has the potential to improve the quality of living in depressed in- and out-patients.

### Conclusions and Implications for Future Research

While there is variation in which aspect of olfaction is altered, our review of the literature found that there indeed is an alteration to olfactory functioning and processing in individuals with depression at baseline and after treatment. Focusing specifically on those studies that employed an interventional methodology, we find that depression and olfaction changed over the course of treatment with an overall improvement in olfactory functioning and depression severity (3, 35, 40, 53). Expansion and replication of these studies is required to confirm the results and if such a change can be found in other forms of depression treatment, such as transcranial magnetic stimulation, electroconvulsive therapy, etc. We suggest that researchers expand an examine this conception further using Sniffin’ Sticks to measure olfactory processing in a larger sample of depressed individuals at baseline and after treatment. We believe a clearer understanding of the relationship between olfaction and depression can be found by adherence and use of our proposed methodology. Olfactory functioning and processing, particularly central processing, are highly integrated with emotion and memory through projections from the OB to the amygdala and hippocampus. Determining if central or peripheral olfactory process as well as OB volume and neurogenesis are impacted in depression allows to generate a clearer and more thorough picture of how depression affects the brain and cognition.

## Author Contributions

All authors contributed substantially to the conception and design, as well as agree to be held accountable for all aspects of the work. HT drafted the article and revised it critically for important intellectual content, as well as gave the final approval of the version to be published. HT and CW contributed to the acquisition, analysis, and interpretation of the data. CW and RM revised the article critically for important intellectual content, as well as gave final approval of the version to be published.

## Conflict of Interest Statement

The authors declare that the research was conducted in the absence of any commercial or financial relationships that could be construed as a potential conflict of interest.
