# Supplementary material for: Olfactory Functioning and Depression: A Systematic Review
Source: Front Psychiatry. 2017 Sep 28;8:190. doi: 10.3389/fpsyt.2017.00190 (PMC5627007; doi:10.3389/fpsyt.2017.00190)
Supplement: Supplementary file 1 [file table_1.docx]

Supplementary Material Appendix

1. Full Review Literature Table

A. Full Review Literature Table

*Supplementary table 1.* Full review of the fifteen manuscripts selected and assessed for quality. All manuscripts examined the relationship between depression and olfactory functioning in patients with depression as the primary diagnosis. Bipolar depression and seasonal affective disorder were both included diagnoses under the umbrella of depression. The following table outlines the sample size, depression measures employed including the version of the *Diagnostic and Statistical Manual* used to define depression, the type of olfactory test used, the specific aspect of olfaction measured, if and what type of intervention/treatment for depression was included as part of the methodology, and the general conclusions with relevant statistics. For short forms, see the bottom of the table.

| Source | **n** | **Age Range and Gender Ratio** | **Depression**  **Measure** | **Olfactory Test(s)** | **Olfactory Measures** | **Treatment/**  **Intervention** | **Conclusion** |
| --- | --- | --- | --- | --- | --- | --- | --- |
| Atanasova et al., 2010 | 30 depressed  30 controls | Age Range: Not Stated  Mean Age: 34.6  Gender Ratio: 12 Female, 18 Male | DSM-IV  MADRS | Two odorants (vanillin & butyric acid) at 3 concentrations each, 9 combination of the odorants and 2 control | Odour hedonic valence  Discrimination  Identification |  | Depressed judged the unpleasant unmixed stimulus as more unpleasant (p<0.05) and more intense while the pleasant was perceived as less intense (p>0.05). MADRS score correlated positive with the probability of identifying the butyric acid (r=0.481, p=0.005) and negatively with the probability of identifying vanillin (r=-0.395, p=0.019). |
| Clepce et al., 2010 | 37 current depressive episode  17 remitted  37 control | Age Range: 23 – 71  Mean Age: 47.52  Gender Ratio: 21 Females, 16 males | DSM-IV  BDI  SHPS | Sniffin’ Sticks | Identification  Hedonics  Intensity | Pharmaological therapy | Those in the current depressive state had significantly lower identification scores compared to the remitted state (p=0.0047) and when compared to the control (12.081 ±0.327 vs 13.014 ± 0.277; F=4.686, p=0.034). No significant difference between patients (depressed or remitted) and controls in hedonic and intensity ratings. |
| Croy et al., 2014 | 27 depressed (female only)  28 control (female only) | Age Range: 22 – 59  Mean Age: 38.5  Gender Ratio: 27 Females, 0 males | BDI  HAM-D | Sniffin’ Sticks | Threshold (sensitivity)  Discrimination  Identification | Psychotherapy | Before psychotherapy, all patients demonstrated reduced scores on all olfactory tests; only the odour discrimination test had a significant difference (t[54]=2.1, p=0.037). No significant group difference occurred after psychotherapy. |
| Gross-Isseroff et al., 1994 | 9 depressed  16 controls | Age range: 34-67  Mean Age: 49.0  Gender Ratio: 8 Females, 1 Male | DSM-III-R  HAM-D | Three-way forced choice of target scent (androstenone or isoamyl acetate) at different concentrations | Threshold | Pharmaological therapy | Threshold for isoamyl acetate was lower but only after 42 days of treatment were the results significantly lower than the control group threshold (F_1,16_=5.95, p=0.03)(t(16) =2.97, p=0.009). No significant effect was found regarding threshold for androstenone at any time point across treatment. There was no correlation between olfactory sensitivity (threshold) and severity of illness. |
| Hardy et al., 2012 | 20 bipolar disorder  44 control | Age Range: 20 – 53  Mean Age: 31.1 (males), 35.6 (females)  Gender Ratio: 15 Females, 5 M ales | DSM-IV  DIGS  PANSS  YMRS | STT  UPSIT-40 | Threshold  Identification |  | No significant difference between the patient and control groups were found in either the threshold or identification. |
| Kopala et al., 1994 | 21 depressed  77 control | Age Range: 21-56  Mean Age: 37.0  Gender Ratio: 13 Females, 8 Males | DSM-III-R | UPSIT-40 | Identification |  | No significant difference in olfactory identification between the depressed and control groups. |
| Lahera et al., 2016 | 39 euthymic bipolar disorder  30 control | Age Range: 18 – 70  Mean Age: 46.82  Gender Ratio: 22 Females, 17 Males | DSM-IV-TR  HAM-D  YMRS | UPSIT-40 | Identification |  | There was a significant odor identification difference between bipolar and control groups (t=3.56, p=0.001) with controls answering more items correctly than the bipolar disorder group (31.85 ±3.6 vs. 27.58 ±6.7). |
| Lombion-Pouthier et al., 2006 | 49 depressed  58 control | Age Range: 20 - 60  Mean Age: 43.4  Gender Ratio: 35 Females, 14 Males | DSM-IV  BDI | Test Olfactif | Threshold  Detection and Identification  Hedonics and Intensity |  | There was a significant difference in olfactory sensitivity between the depressed and control (4.2 ±1.38 vs. 3.66 ±1.36; F_3,141_ = 5.692, p=0.001). The post hoc comparisons (Fischer PLSD) further substantiated the difference (p<0.035). The identification, detection, intensity, and hedonics were not significantly different between the control and depressed groups. |
| Naudin et al., 2012 | 18 depressed  18 clinically improved  54 conctols | Age Range: 20-74  Mean Age: 50.1  Gender Ratio:12 Females, 6 Males | DSM-IV  MADRS | Eight odorants presented successively for familiarity, pleasantness and identification. Two odorants at different concentrations and different combinations | Hedonics  Familarity  Discrimination/Intensity  Identification |  | No significant difference between the groups in identification, discrimination, or familiarity test. Only the unpleasant odorants, butyric acid, was noted as significantly more unpleasant by the depressed compared to controls (p=0.003). |
| Negioas et al., 2010 | 25 depressed  22 control | Age Range 21 – 55  Mean Age: 36.86  Gender Ratio: 17 Females, 4 Males | DSM-IV  BDI | Sniffin’ Sticks | Threshold (sensitivity)  Discrimination  Identification |  | No significant difference between depressed and controls on discrimination and identification; however there was a significant difference in threshold scores which were lower in depressed compared to controls for the right nostril(6.97±2.37 vs. 8.54 ±2.19; t[37]=2.15, p=0.038), left nostril (6.84 ±2.84 vs. 8.40 ±1.68; t[37]2.11, p=0.041), and best nostril (7.56 ±2.67 vs. 9.14 ±1.89; t[37]=2.21, p= -.032). |
| Pause et al., 2001 | 24 depressed (18 participated at time 2)  24 control | Age Range: Not Stated  Mean Age: 48.4  Gender Ratio: 15 Females, 9 males | DSM-IV  BDI | Ten odors were presented for valence and intensity. Threshold was obtained using two odorants at varying concentrations in a staircase detection procedure. | Threshold (Sensitivity)  Intensity | In-patient treatment involving psychotherapy and pharmacological intervention | At time 1, depressed patients had strongly reduced sensitivity (F_1,45_=8.59, p=0.005) and this effect in eugenol was significantly negatively correlated with BDI scores (r=-0.344, p=0.009). There was no significant difference in intensity scores between the groups and no correlation between phenyl-ethylalcohol sensitivity and the BDI. At time 2, no significant difference was found and the BDI score could not accurately predict olfactory sensitivity with either phenyl-ethylalcohol or eugenol. |
| Postolache et al., 2002 | 14 seasonal affective disorder (SAD)  16 control | Age Range: 27 – 66  Mean Age: 42.3  Gender Ratio: 7 Women and 7 Males | DSM-IV  SIGH-SAD | Phenyl ethyl alcohol presented in a staircase paradigm | Threshold |  | Those in the SAD group had significantly lower detection thresholds than the controls (F_1,25_=9.2, p=0.006). A nonsignificant trend for larger differences between patients and controls in the summer than winter (F_1,26_=3.25,p=0.08) was also observed. |
| Swiecicki et al., 2009 | 20 recurrent depressive disorder  21 bipolar affective disorder  5 first lifetime episode of depression  30 control | Age Range: 18-70  Mean Age: 38.2  Gender Ratio: 30 Females, 16 Males | DSM-IV  HAM-D  AUDIT  BDI | Sniffin’ Sticks | Threshold  Identification  Anhedonia |  | No differences between the depression and control groups nor within the depression groups were found with regards to olfactory threshold, identification, and the number of odors rated as unpleasant, neutral, or pleasant. There was also no correlation between depression severity and the number of olfactory stimuli rated as pleasant, neutral, or unpleasant. |
| Warner et al., 1990 | 6 depressed  8 control | Age Range: 38-50  Mean Age: 37.0  Gender Ratio: 0 Females, 6 Males | RDC | UPSIT-40 | Identification |  | No significant difference between the depressed and control groups in the identification scores were found. |
| Zucco et al., 2011 | 12 mild depressed  12 severe depressed  12 control | Age Range: 23 – 58  Mean Age: 41.3  Gender Ratio: 12 Females, 12 Males. | DSM-IV | 10 odorants presented along with 4 other odorants to determine which was previously presented. 10 odorants presented with 4 verbal options for naming the target. | Identification  Recognition |  | The severe MDD group performed significantly worse (8.21±1.05) on the identification and recognition tasks compared to both the mild depressed (9.33±0.65) and control group (9.58±0.51)(F_2,33_= 15.89, p<0.001). |

DSM, Diagnostic and Statistical Manual; HAM-D, Hamilton Depression Rating Scale; MADRS, Montgomery-Asberg Depression Rating Scale; BDI, Beck Depression Inventory; SHPS, Snaith-Hamilton Pleasure Scale; DIGS, Diagnostic Interview for Genetic Studies; PANSS, Positive and Negative Syndrome Scale; UPSIT-40, University of Pennsylvania Smell Identification Test, 40-item test; YRMS, Young Mania Rating Scale; STT, Smell Threshold Test; SIGH-SAD, Structured Clinical Interview Guide for the Hamilton Depression Rating Scale, Seasonal affective disorder version; AUDIT, Alcohol Use Disorders Identification Test; RDC, Research Diagnostic Criteria.
